# Effect of Confinement on Photophysical Properties of P3HT Chains in PMMA Matrix

**DOI:** 10.1186/s11671-017-2270-y

**Published:** 2017-08-29

**Authors:** Oleg P. Dimitriev

**Affiliations:** 0000 0004 0385 8977 grid.418751.e V. Lashkaryov Institute of Semiconductor Physics, Natl. Acad. of Sci. of Ukraine, Prospect Nauki, 41, Kiev, 03028 Ukraine

**Keywords:** Poly(3-hexyl-thiophene), Poly(methyl methacrylate), Photoluminescence, Disorder

## Abstract

The influence of arrangement of poly(3-hexylthiophene) (P3HT) chains embedded into poly(methyl methacrylate) (PMMA) matrix on photophysical properties, such as electronic absorption spectrum, band gap, and photoluminescence quantum yield, of the formed P3HT aggregates have been studied. It has been found that variation of P3HT fraction in PMMA matrix from 25 to 2 wt% is accompanied with the increasing quantum yield of photoluminescence, red shift of the band gap, and structural change of P3HT crystallites. The above changes are accompanied with disruption of the continuous network of P3HT fraction into smaller P3HT particles with size ranged from several microns to several tens of nanometers. The results are interpreted in terms of the changing intermolecular packing and reduced intramolecular torsional disorder. It is discussed that the most contribution to the above changes comes from P3HT molecules at the interface of P3HT cluster and PMMA environment.

## Background

Photophysics of collapsed coils and nanoscale confined systems of conjugated polymers has attracted considerable interest during last decade [1–4]. Particularly, the processes of exciton generation, radiative recombination, and photogenerated charge transfer in poly(3-hexylthiophene) (P3HT) nanoscale aggregates and crystallites have a direct impact on performance of organic solar cells where this polymer is used as an active component. It was shown that the nature of emission in isolated P3HT molecules and P3HT aggregates is different. The molecular emission normally originates from a common intrachain exciton state corresponding to the relaxed chain with reduced torsional disorder [5]. The emission spectrum of P3HT aggregates also originates from a common emitting state, but corresponding to the interchain singlet exciton that has fell down by single or multiple energy transfer steps to the domain with the lowest energy [6]. Quantum yield (QY) of photoluminescence (PL) of ordered lamellae structure in P3HT films is strongly suppressed as compared to the free molecules in solutions due to interchain delocalization and dissipation of excitons in the condensed material [7]. On the other hand, QY can be enhanced by control of temperature [8] or regioregularity of P3HT chains [9]. It was shown, for example, that regioregular P3HT films has an order weaker optical transitions as compared to films of regiorandom P3HT due to a larger interchain contribution for the lowest exciton in lamellae compared to the intrachain character of exciton in regiorandom P3HT [9]. Therefore, developing simple and effective strategies to manipulate the optical properties of conjugated macromolecules through changes in their intramolecular design and intermolecular assembly and ordering has significant potential for gaining further understanding of this interesting class of materials but also for their widespread application in organic electronics.

The goal of this work is to show how the changed arrangement of P3HT chains influences physical properties, such as electronic absorption spectrum, band gap, and emission QY of P3HT nanoscale particles. One promising strategy that enables one to tune photophysical properties of conjugated polymer films is blending with the other inert polymer. It is known that in the case of P3HT, its optical properties can be readily influenced by the presence of a suitable host medium. For example, Lee et al. showed that the optical transition energies in absorption and emission experiments of P3HT nanoparticles are affected by a hydrothermal (polar) treatment with deionized water at temperatures of up to 150 °C in an autoclave [10]. Hellmann et al. showed that blending of P3HT with the polar poly(ethylene oxide) (PEO) leads to the increased 0-0 oscillator strength as well as to a considerable shift of the optical absorption spectrum by 0.1 eV [11]. In addition, Kim et al. observed similar changes in the optical properties of electrospun P3HT nanofibers after blending the P3HT and PEO and spinning them from polar solvent mixtures [12]. Other studies have demonstrated a minor redshift in the optical absorption spectrum of P3HT films by blending with poly(ethylene glycol) without the need for additional polar solvent additives [13]. Thus, the above experiments have indicated that the photophysical properties of P3HT can be readily manipulated by processing means. Although above studies showed significant influence of the host environment on band gap of P3HT aggregates, the changes in emission QY have been paid less attention. For example, Kanemoto et al. have showed that PL of conjugated polymers can be enhanced in the solid state by dilution using moderate inert polymers such as polypropylene [14]. However, this effect was achieved by conversion of aggregates to the molecular form of the conjugated polymer.

Here, we demonstrate that blending conjugated polymer P3HT with polar poly(methyl methacrylate) (PMMA), where P3HT particles of micro- and nanoscale are formed, induces systematic changes in physical characteristics of P3HT aggregates. We show that as the weight ratio of P3HT to PMMA decreases, the P3HT fraction demonstrates the redshifting band gap, the improvement in ordering, and the enhancement in QY of emission. We show that these changes very likely are due to planarization of the conjugated polymers’ backbone in the presence of PMMA under the action of hydrophobic forces from the host material.

## Methods

### Sample Preparation

Initial stock solution of regioregular P3HT (~ 93% RR, 99,995% trace metal basis, with number-average molecular weight (M_n_) in the 15–45 kDa range, Sigma-Aldrich) was prepared with concentration of 1.0 wt% in chlorobenzene (CB). Binary mixtures of P3HT and PMMA were prepared by addition of a necessary amount of poly-(methyl methacrylate) (PMMA, average molecular weight (M_w_) of 120 kDa, Sigma-Aldrich) to P3HT solution in CB followed by treatment in the ultrasound bath for 30 min. Films have been prepared by spin-coating onto glass substrates at 1500 rpm for 30 s.

For transmission electron microscopy (TEM) studies, the film was scraped away to the vessel with acetone, which then stayed several hours to ensure that all PMMA was completely dissolved releasing P3HT aggregates which are practically insoluble in acetone (solubility of P3HT in acetone is less than 0.1 mg/mL [15]). A small amount of the solution was drop-cast onto the TEM carbon grid followed by evaporation of acetone. PMMA solution in acetone was drop-cast on a separate grid in order to get images of the neat PMMA samples.

### Spectroscopy Measurements

Absorption spectra were measured using a SPECORD M40 and an OLIS Cary 14 double beam spectrophotometers. Bare glass plate served as a reference. Fluorescence spectra were collected using a SPEX Fluorolog 1680 double spectrometer, with a Xe lamp as a light source. The excitation wavelength was selected at 468 nm. Absorption spectra are given below as normalized to their maximum in order to compare their spectral features, and the PL spectra are given corrected for the sensitivity of the registering system and normalized to the sample absorption at the excitation wavelength, i.e., the PL spectra are presented in terms of the relative QY of the sample emission.

The transient absorption (TA) pump–probe measurements were performed using a Ti:sapphire laser system. The excitation was set at a wavelength of 410 nm. The TA measurements were carried out with the pump (with repetition rate of 1 kHz and pulse duration of ~ 100 fs) and a white light continuum generated by a sapphire crystal as the probe. The pump beam was modulated mechanically at exactly half the repetition rate of the CPA system (500 Hz), and Δ*T*/*T* or ΔOD was detected with a phase sensitive technique using lock-in amplifiers. The polarization of the pump beam was at the magic angle (54.7°) relative to that of the probe beam. The measured fractional transmission signals, i.e., TA, are given by TA = −Δ*T*/*T*= (*T*_on_-*T*_off_)/*T*_off_, where *T*_on_ denotes the probe transmission with pump on, and *T*_off_ the probe transmission with pump off. The obtained spectra were rectified by wavelength calibration procedure.

### Microscopy Measurements

Morphologies of the samples were studied both by optical microscopy and TEM. Optical micrographs of the samples were taken using optical microscope ULAB XY-B2 equipped with a photo-camera and a computer. TEM studies were performed using JEOL JEM-1400 instrument operating at 80 kV.

## Results

### Photophysical Studies

**Fig. 1:**
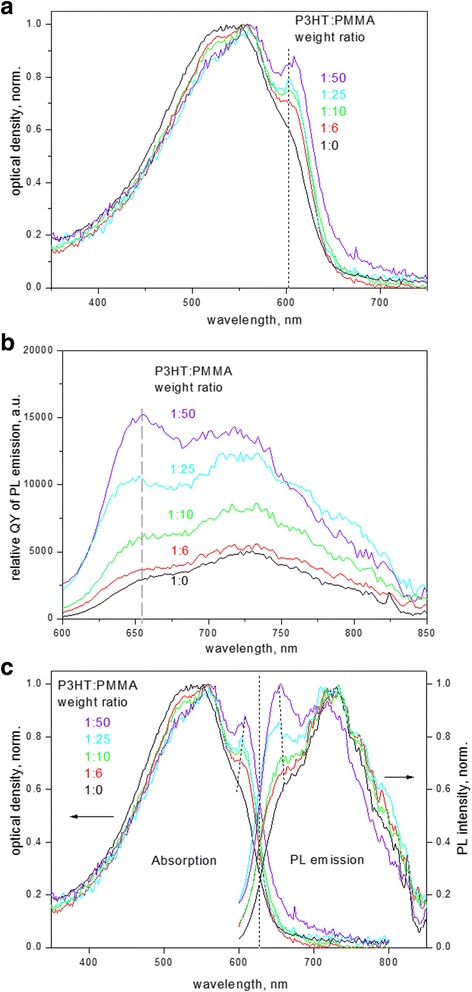
**a** Normalized electronic absorption spectra, **b** PL spectra (in terms of relative QY)  and **c **comparison of normalized absorption and PL spectra of P3HT-PMMA composite films with different P3HT:PMMA weight ratios

Electronic absorption spectra of P3HT composite films (Fig. 1a) demonstrate a typical onset of the absorption from about ~ 650 nm (1.9 eV) corresponding to the band gap of P3HT crystals, followed by vibronic replicas at ~ 605, 560, and 525 nm which are related to fundamental (0-0) transition,(0-1) and (0-2) sidebands, respectively. There is a gradual evolution in spectra as the weight ratio of P3HT to PMMA decreases. First, the amplitude ratio of (0-0) to (0-1) absorption increases. Second, the absorption spectra show narrowing from the short-wavelength side of the spectrum; this region is normally attributed to the absorption of disordered molecules in an amorphous state since molecular absorption of P3HT in diluted solutions is observed just around ~ 460 nm; the above changes therefore indicate a decrease of the disordered amorphous fraction in the sample [6, 8]. Third, the absorption maximum related to the (0-0) transition is gradually shifted from 602 to 608 nm; the band gap, as calculated from the intersection of the tangent line to the absorption edge and the abscissa axis, becomes also red shifted from 1.92 to 1.89 eV as P3HT to PMMA ratio in the composite films decreases.

PL emission spectra (Fig. 1b) demonstrate a Stokes shift by ca. 0.15 eV, and the spectra have a similar behavior to the absorption ones, with the mirror sequence of the sidebands. The shape of the PL spectra and particularly the ratio of (0–0) to (0–1) band intensities are also dependent on the fraction of P3HT in PMMA matrix. The above changes correlate well in both electronic absorption and PL spectra and indicate the extent of ordering in P3HT films [16, 17]. The amplitude ratio of (0-0) to (0-1), which is smaller than 1, is characteristic of H-aggregates coexisting with non-aggregated chain sequences [18, 19]. In addition, both absorption and PL spectra of the films show an increasing intensity of the first maximum in respect to the sidebands (Fig. 1a, b) as P3HT to PMMA ratio decreases. The relative increase in intensity of the (0-0) transition in spectra evidences in favor of rearrangement of P3HT chains in films. The intensity ratio of (0-0) to (0-1) bands is related to the free exciton bandwidth *W* as well, whose non-zero magnitude reflects the extent of disorder in the polymer chains [16] and which can be calculated by using Eq. (1) below under assumption that a Huang-Rhys factor is equal to unity [20, 6]:

1 $$ \frac{A_{0-0}}{A_{0-1}}\approx \frac{n_{0-1}}{n_{0-0}}{\left(\frac{1-\frac{0.24W}{E_p}}{1+\frac{0.073W}{E_p}}\right)}^2 $$

where *n*_0−i_ is the real part of the refractive index at the 0–i peak and *Ep* is the phonon energy of the main oscillator coupled to the electronic transition. In Eq. (1), a refractive index ratio is ∼ 0.97 [6], and the main intramolecular vibration *E*_*p*_ is dominated by C=C symmetric stretch at 0.18 eV [21]. In more ordered polymer chains, the Coulombic interchain coupling is weaker, which leads to narrowing exciton bandwidth. The non-zero exciton bandwidth affects the energy position of the first absorption maximum in P3HT as well, as excitation takes place to the upper level of the exciton band, whereas emission occurs from the low level of the band, respectively. The influence is as follows: the wider the exciton bandwidth, the larger the separation of the first PL emission maximum (assigned as the 0-0 transition in P3HT aggregates [20]) and the first absorption maximum; this tendency is indicated by the dashed lines in Fig. 1c.

**Fig. 2:**
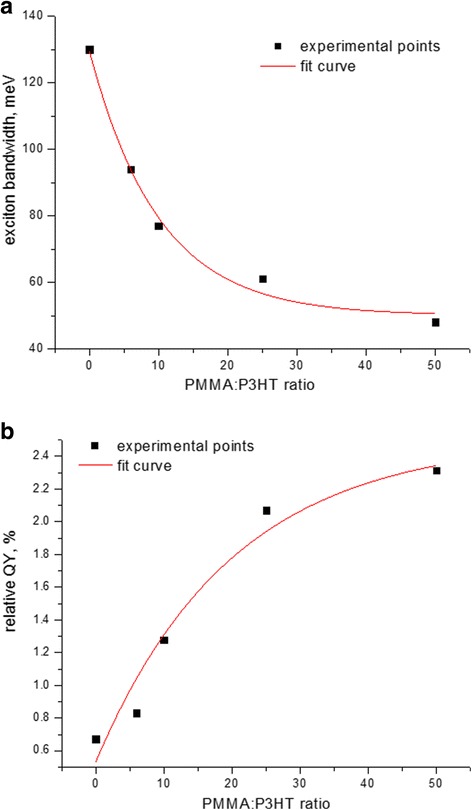
**a** Exciton bandwidth and **b** relative QY of emission of P3HT-PMMA composite films as a function of PMMA:P3HT weight ratio, assuming that the QY for the neat P3HT film is about 0.5% [25]

Exciton bandwidth demonstrates narrowing as the ratio of P3HT to PMMA decreases (Fig. 2a), accompainied by increasing QY of P3HT emission by a factor of four (Fig. 2b) . Such a behavior should correspond, on one hand, to P3HT chain ordering. On the other hand, a decrease in the exciton bandwidth is related to the increasing intrachain and decreasing interchain correlation [21], implying a simultaneous increase in the intrachain order and conjugation length of the polymer backbone and a decrease in quantity of chains participating in π − π interaction over which the exciton is delocalized, theoretically approaching a zero exciton bandwidth for an ideally ordered long chain [22]. However, the dependence of the exciton bandwidth on PMMA:P3HT ratio in Fig. 2a can be fitted by an exponent with an offset. The offset indicates that the bandwidth does not approach zero; instead, it goes to some saturation level of 45 ± 5 meV (Fig. 2a). That means that there is a limiting non-zero size of ordered P3HT aggregates in P3HT matrix that gives rise to interchain exciton independent on how small is the fraction of P3HT in PMMA matrix. This finding is related to a strong property for regioregular polythiophenes to self-assemble [23] and to form highly ordered crystalline domains whose characteristic size can be as small as ~ 10 nm [24, 8]. However, our calculated limiting value of the exciton bandwidth is somewhat larger compared to that observed for P3HT crystallites obtained from other poor solvents such as mesitylene or isodurene (*W* ~ 30 meV [21]); this discrepancy can be explained in our case by the presence of a good solvent, i.e., CB, during film formation from the ternary P3HT-PMMA-CB system [25].

**Fig. 3:**
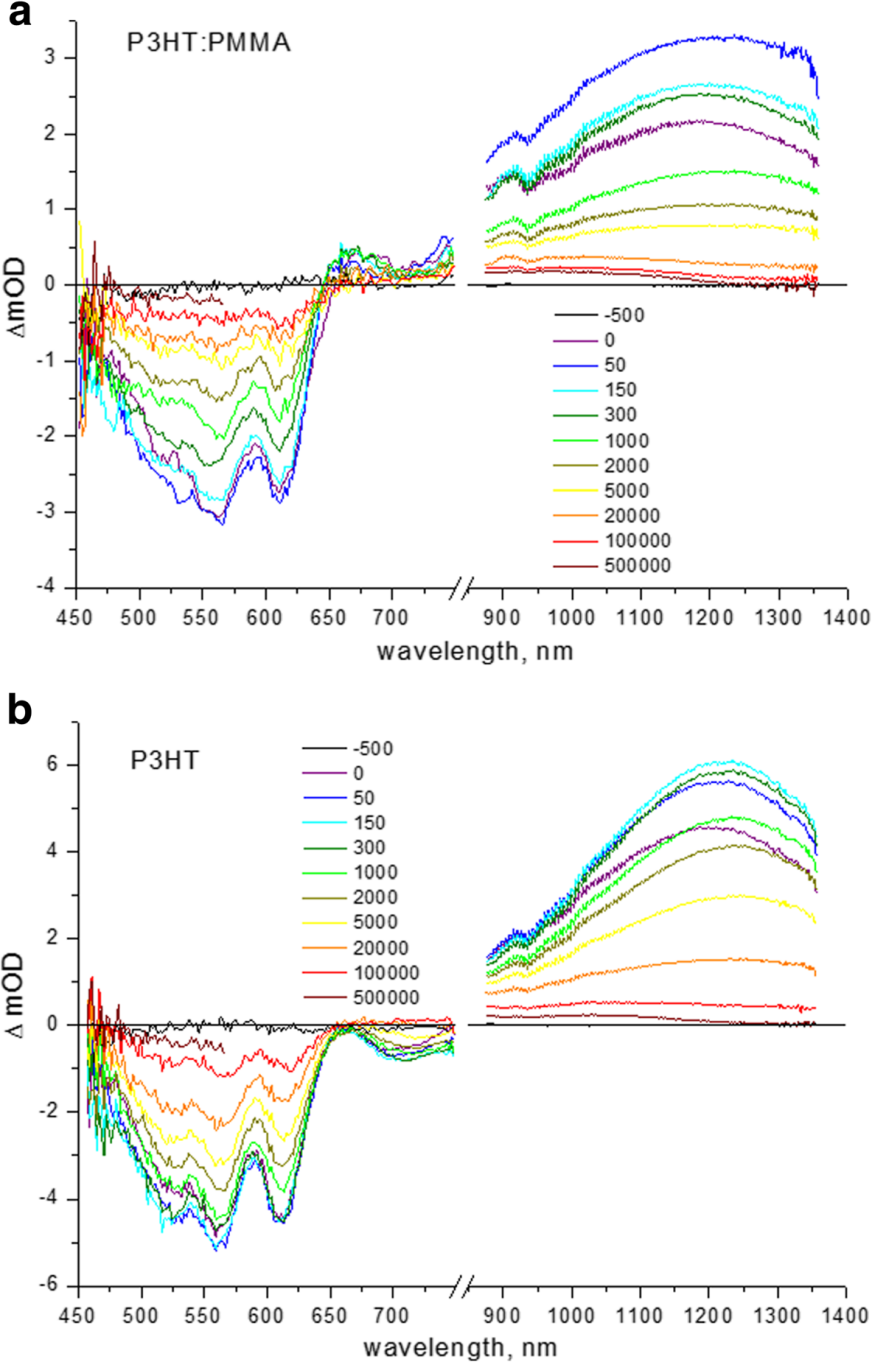
TA spectra of **a** P3HT:PMMA (1:50 weight ratio) and **b** neat P3HT spin-coated films. Time delays are indicated in femtoseconds in the vertical bars

TA spectroscopy provided additional evidence on ordering of P3HT aggregates in PMMA matrix. Comparison of TA spectra of the neat P3HT and P3HT:PMMA composite films are shown in Fig. 3. The typical spectrum consists of two negative bands, one of them is the ground state bleaching (GSB) in the region of 530–630 nm, indicative of bleaching of 0-1 and 0-0 absorption of P3HT, and the other band at ~ 700 nm is indicative of stimulated emission (SE). The positive bands in spectra at ~ 660 and ~ 950 nm are characteristic of polaron absorption delocalized in ordered crystalline domains and localized in disordered amorphous domains, respectively [25–27]. The band at ~ 1200 nm is normally assigned to singlet exciton TA in P3HT [7, 28–30]. The characteristic difference in the above spectra (Fig. 3) is that the P3HT chains in PMMA matrix demonstrate the obvious delocalized polaron absorption at ~ 660 nm indicative of the presence of substantial crystalline ordered regions of P3HT, whereas pronounced SE at ~ 700 nm in the neat P3HT film is characteristic of intrachain excitons in disordered P3HT chains [8].

Analysis of the (0-0) transition and (0-1) vibronic sideband in TA spectra reveals different relative dynamics of relaxation in the neat P3HT and composite P3HT-PMMA films (Table 1). In the neat P3HT film, the (0-1) vibronic sideband decays slower as compared to relaxation of the (0-0) transition, indicating the major lifetime (with contribution of ~ 60%) of 7.0 ps for (0-1) transition versus 5.3 ps for (0-0) transition, respectively. In P3HT-PMMA composite, the major lifetime (~ 73%) is shorter and similar for the both (0-1) and (0-0) transitions, being ~ 1.8 ps, while lifetime of the minor component (~ 27%) is slower for the (0-0) transitions (approximately 300 vs. 200 ps, respectively), providing a faster relaxation of the (0-1) vibronic sideband overall (Fig. 3). The fast component of relaxation of the order of picoseconds is characteristic of torsional relaxation leading to planarization of P3HT chain upon photoexcitation [31, 32], whereas the slow component is characteristic of the lifetime of nonfluorescent excitons that are probed by TA measurements [7]. The different behavior of relaxation in the GSB region for the neat P3HT and P3HT-PMMA composite films points out in favor of faster planarization of P3HT chains in the composite sample upon photoexcitation; that implies that the chains have already been partly planarized in the ground state, i.e., they have less torsional disorder in the composite film than in the neat P3HT film. On the other hand, the exciton TA demonstrates faster decay in P3HT-PMMA composite as well, with typical times of 0.6 ps (68%) and 19 ps (32%) vs. 2 ps (51%) and 40 ps (49%) for the neat P3HT film, respectively (Table 1). The above ultrafast component of decay can be assigned to the intrachain exciton energy transfer from high-energy to low-energy site [33] and the slower component to the isoenergetic energy transfer after the fast exciton migration has occurred [34]. It seems reasonable to suggest that the intrachain exciton migration proceeds faster in more ordered chain, free of torsional disorder, again confirming that P3HT chains have a better ordering in the composite sample.

**Table 1:** Decay time constants and amplitudes of TA spectra of P3HT and P3HT:PMMA films in the GSB and exciton regions; the dynamics is fitted by two-exponential decay *OD*(*t*) *= A*_*1*_ *exp*(*−t/t*_*1*_) *+ A*_*2*_ *exp*(*−t/t*_*2*_)

Film composition	0-1 (560 nm)		χ^2^	0-0 (610 nm)		χ^2^	Exciton (1240 nm)		χ^2^
Neat P3HT	7.0 ps (60%)	295.1 ps (40%)	0.976	5.3 ps (63%)	424.7 ps (37%)	0.977	2.0 ps (51%)	40.2 ps (49%)	0.996
P3HT:PMMA (1:50 weight ratio)	1.8 ps (73%)	202.6 ps (27%)	0.980	1.9 ps (74%)	299.0 ps (26%)	0.979	0.6 ps (68%)	18.9 ps (32%)	0.990

### Morphology Studies

**Fig. 4:**
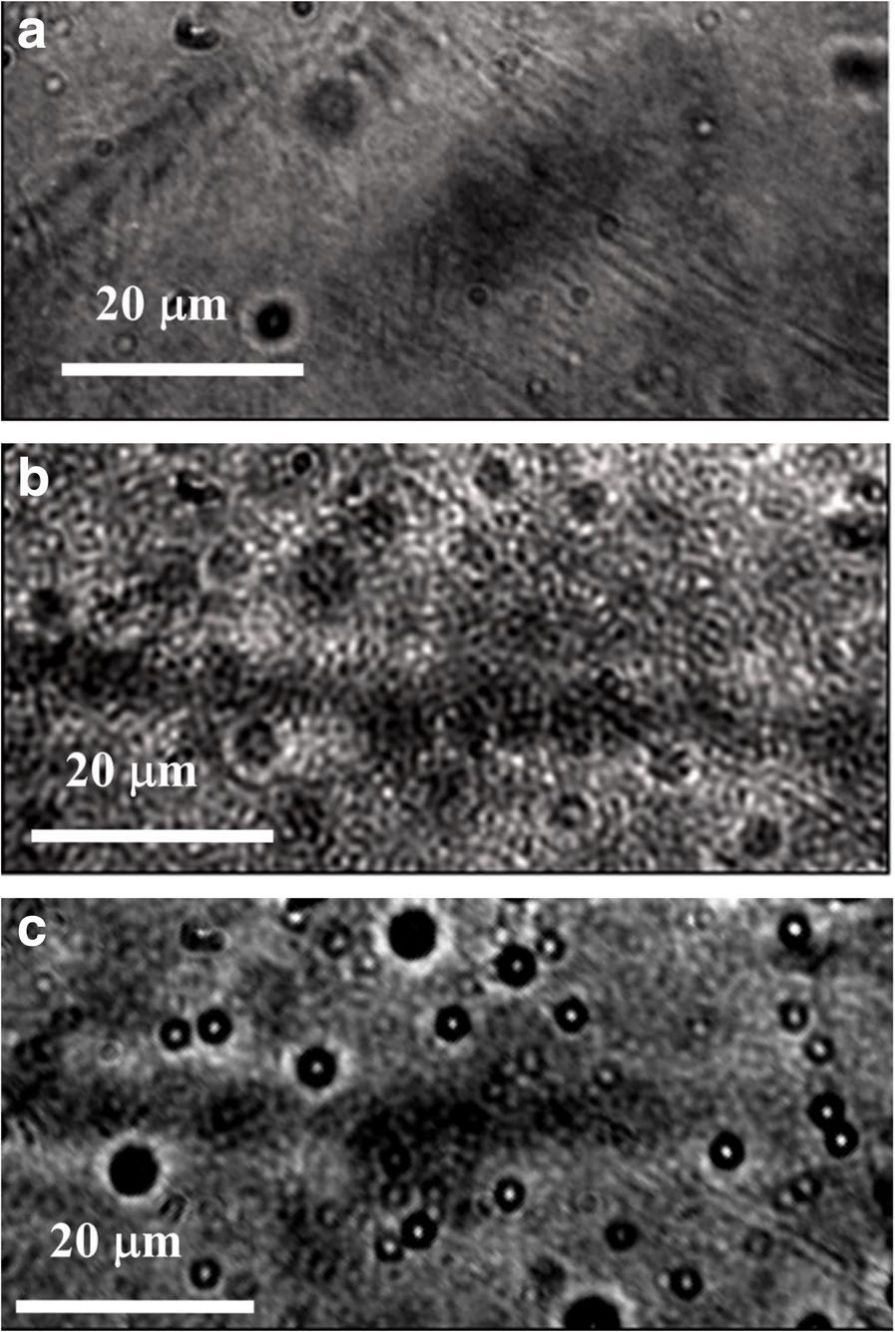
Optical micrographs of **a** neat P3HT and **b**, **c** P3HT:PMMA films with **b** 10 wt% and **c** 2 wt% of P3HT in PMMA

**Fig. 5:**
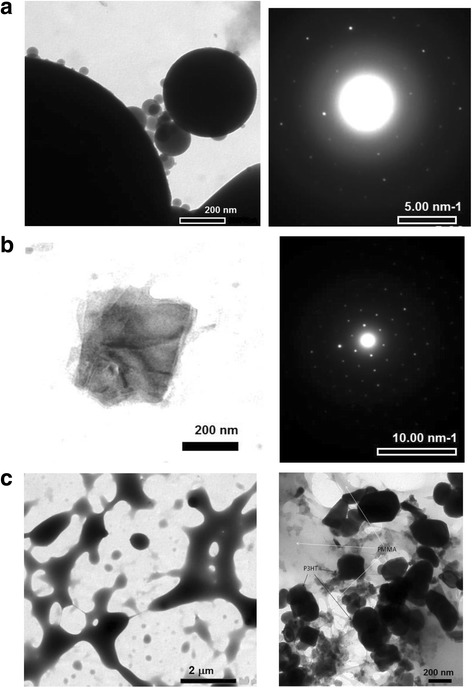
TEM images of **a** spherical particles of P3HT, **b** crystalline domain of P3HT, and their corresponding SAED patterns to the right of the images; **c** neat PMMA (*right image*) and P3HT-PMMA blend (*left image*) are given for comparison

Microscopy studies allowed us to observe the size distribution of P3HT fraction in PMMA matrix as P3HT:PMMA ratio changes. First, the composite P3HT:PMMA films reveal a highly structured morphology, pointing out that phase separation of P3HT and PMMA takes place, in contrast to the neat P3HT film whose morphology is relatively smooth (Fig. 4). However, at relatively high concentration of P3HT (~ 10 wt% and more), P3HT fraction is continuous and forms a percolation network in PMMA matrix. At small concentration of P3HT, the polymer fraction transforms to separate P3HT particles of micron and submicron size. TEM image (Fig. 5a) shows that the particles can be as small as ~ 30 nm and have practically an ideal spherical form. The spherical shape of the particles allows us to suggest on repulsive forces between P3HT and PMMA, where amorphous (“liquid-like”) phase of P3HT tends to separate from PMMA matrix into compact particles possessing a minimal surface. Therefore, a sufficient fraction of the amorphous phase should be suggested in P3HT particles. Selected-area electron diffraction (SAED) pattern (Fig. 5a, on the right) shows superposition of both amorphous and crystalline features of the particles. Separate highly crystalline non-spherical domains have also been found in the sample (Fig. 5b). Therefore, the spherical P3HT particle is suggested to consist of a crystalline core surrounded by an amorphous phase of P3HT. Rahimi et al. found that even highly ordered single crystals of P3HT are surrounded by a fraction of about 12% molecules that adopt solution-like conformation and that the characteristic thickness of the amorphous layer is about 10 nm [35]. Assuming that the amorphous layer of a similar thickness is formed around a crystalline core, it is easy to understand that a particle that has a size of ~ 30 nm can easily adopt a spherical shape due to such an amorphous shell.

### Assignment of the Crystal Structure

**Fig. 6:**
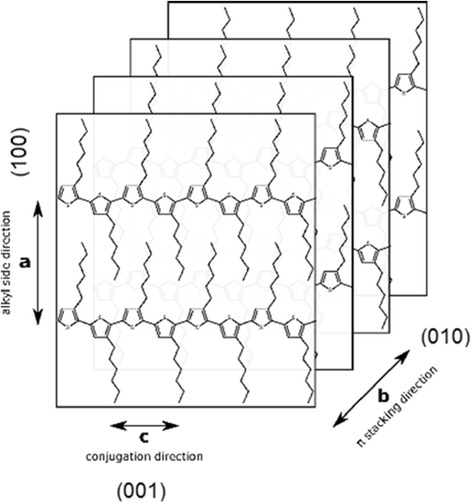
Scheme of the crystalline structure of P3HT

In general, the thermodynamically favored texture is assumed to be formed by edge-on oriented chains of P3HT [36, 37]. This structure is obtained under conditions close to the equilibrium realized for slow film-formation methods such as drop-casting [38, 39], dip-coating, and spin-coating from high-boiling point solvents [40]. In samples prepared on TEM grids, the edge-on orientation is preferred due to hydrophobic grid surfaces (carbon) which prefer interactions with hydrocarbon substituents of P3HT molecules. Such an orientation provides “visualization” of mainly (010) and (001) planes of P3HT lamella crystals in SAED patterns (Fig. 6).

**Fig. 7:**
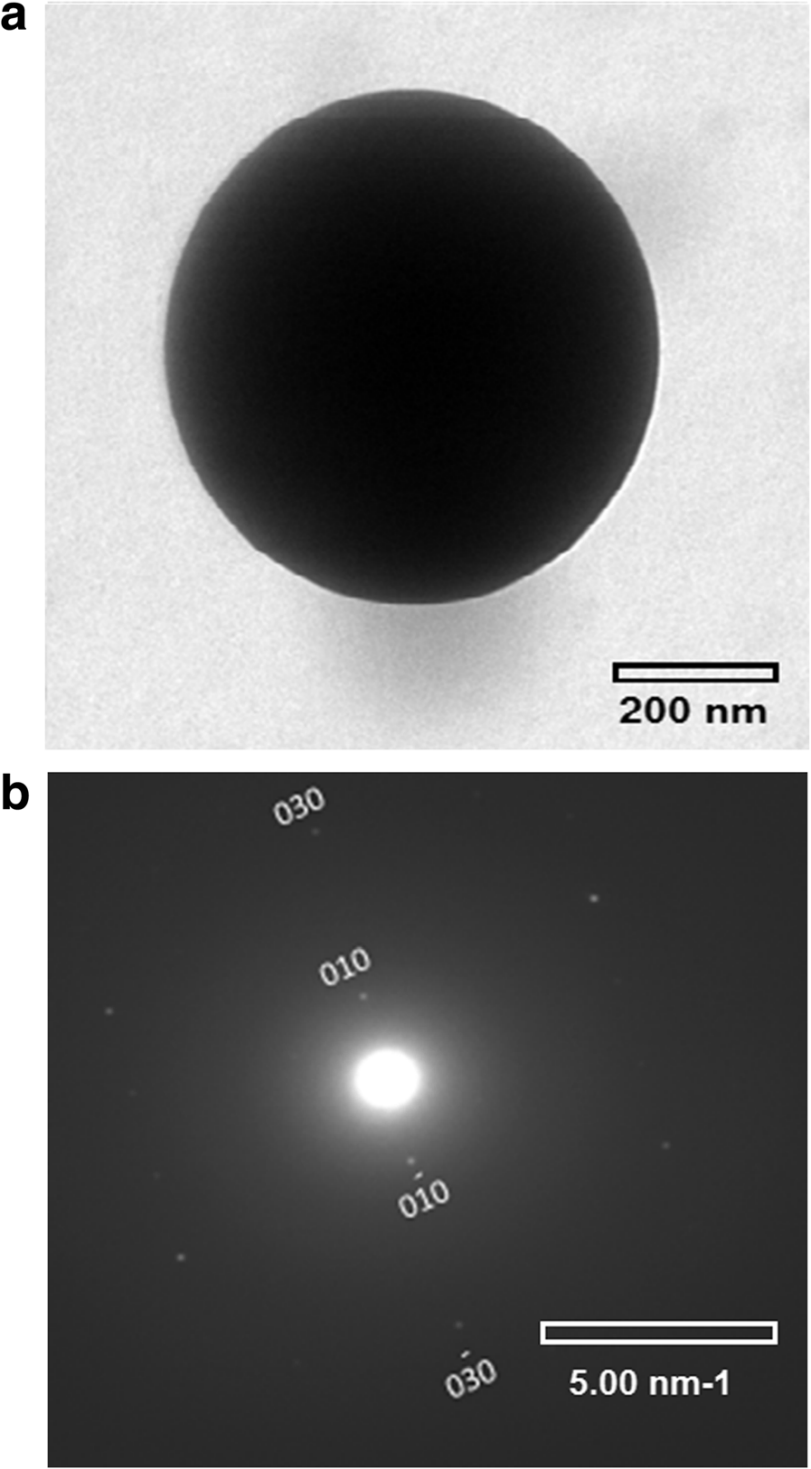
**a** TEM image and **b** SAED of a ball-like P3HT domain in P3HT-PMMA (1:50 weight ratio) blend sample

**Fig. 8:**
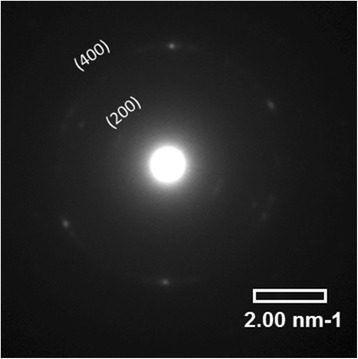
SAED of a P3HT domain in P3HT-PMMA (1:50 weight ratio) blend sample

The following stacking periods of successive polythiophene backbones along the *b* axis were obtained: 0.45 ± 0.1 nm for the ball-like structures (Fig. 5a, Fig. 7) and 0.48 ± 0.1 nm for the separate lamella (Fig. 5b). The obtained values are too large to assign them to the form I of P3HT crystal taking into account that the angle between *a* and *b* axes is usually close to 90° [41]. Moreover, some SAED patterns allowed us to find (h00) diffraction rings (Fig. 8), from where it is possible to determine the inter-stacking distance (the distance between P3HT chains separated by layers of *n*-hexyl side-chains, i.e., along the *a* axis of the monoclinic unit cell, Fig. 6), being 1.23 nm. The obtained distance is characteristic of the crystalline form II. Thus, if we try to attribute the crystals to the form II, we should take into account that the form II crystal has a tilt angle γ = 68° between the *b* axis and the thiophene planes [42], from where one can calculate the short interplanar distances to be 0.417 and 0.445 nm, respectively. The latter value agrees well with the short interplanar distance in crystalline form II (0.44 nm [43]), whereas the former one better corresponds to an intermediate form I’ with the interplanar distance of 0.41–0.42 nm [44].

## Discussion

The major finding of this work is that QY of emission of the P3HT condensed phase can be enhanced not due to disentanglement of tightly packed aggregates with substantial exciton quenching by neighboring molecules into molecular form of P3HT, but by simple reduction of the size of P3HT condensed phase to micro- and nanoparticles. Two main reasons can be considered which are responsible for the above phenomenon: First, the increase in the interface area of P3HT/PMMA, where the interfacial molecules increase their contribution to the emission properties due to increasing surface-to-volume ratio in the diminishing P3HT particles; Second, the changed arrangement of P3HT chains in the crystalline domains as a result of repulsive forces acting from PMMA, which affect more P3HT molecules as the ratio of P3HT to PMMA decreases.

**Fig. 9:**
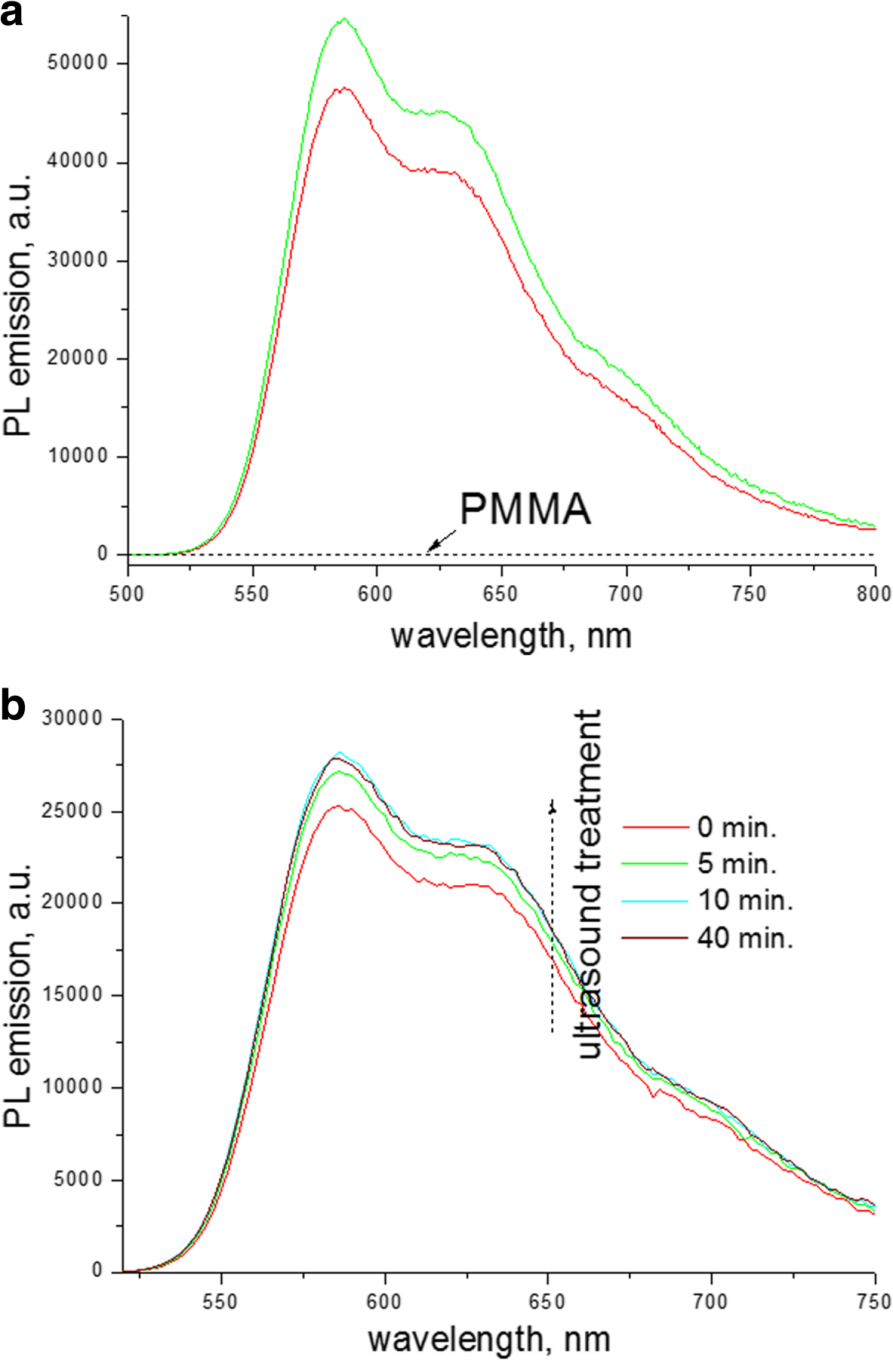
PL emission spectra (λ_exc_ = 468 nm) of P3HT solution (0.01 wt%) in CB: **a** before (*red*) and after (*green*) mixing with solution of PMMA (5.4 wt%), PL spectrum of PMMA is given as well; **b** upon addition of PMMA powder to the neat P3HT solution (*red*) followed by successive treatment of the cuvette in an ultrasound bath and gradual dissolution of PMMA to ~ 3.25 wt%

The first possible reason implies the change in dielectric constant of P3HT environment. Indeed, Hu et al. reported that the replacement of relatively polar solvent with high dielectric constant (ε > 3) by a nonpolar one with low dielectric constant (ε < 3) leads to enhancement in fluorescence QY of P3HT aggregates by almost one order of magnitude [45]. It should be noted that PMMA has ε > 2.8 [46] and in principle, it can be considered to affect QY of PL emission. In order to verify the contribution of this factor, we checked the PL emission of P3HT as the solvent environment was gradually replaced by PMMA molecules (Fig. 9). In the first experiment, the same quantities of the stock solution of P3HT were added to the cuvettes with CB and concentrated solution of PMMA in CB, respectively, where solution volumes were the same (Fig. 9a). In the second experiment, in order to remove possible casual error of the syringe volume that supplies a P3HT solution, PMMA powder was added to P3HT solution and the spectra were measured successively during dissolution of PMMA (Fig. 9b). The both experiments showed a small but distinct increase in the relative QY of emission of  P3HT solution in the presence of PMMA. Therefore, the changes in the dielectric constant of CB (ε ~ 5.6) to that of CB-PMMA mixture and then to the neat PMMA environment in thin films should facilitate enhanced fluorescence QY. This effect in solutions, however, was assessed to be small, inducing an increase in QY of PL by only ~ 14%. On the other hand, in films, the increase in QY of PL was found to be up to ~ 400% (Fig. 2b). Therefore, the relative change in dielectric constant has only an attendant weak effect on enhancement of QY of PL in thin composite films.

The other important factor which can be particularly inferred from the spectral changes of P3HT-PMMA films is the change in mutual arrangement of polymer chains in P3HT domains being in PMMA matrix. It should be noted that P3HT crystals can adopt different forms, i.e., the form I which is being most commonly observed in thin films after annealing [47], or the form II which represents an energetically more stable situation [42]. Form II can be obtained, for example, by synergetic action of hydrophilic polymer matrix and a poor solvent such as water on P3HT chains during film formation [11], and it displays a notable red shift in the absorption spectrum [35]; a similar tendency is observed in our results, showing red shift of the band gap from 1.92 to 1.89 eV (Fig. 1a). Interestingly, the π − π stacking distance reported for P3HT nanofibrillar crystals of form II is relatively large, being from 0.440 nm [43], as compared to the stacking distance found for the form I which is between 0.340 and 0.414 nm [48–50]. At the same time, there is a tighter alkyl side-chain inter-digitation in form II, with the interchain distance (in the direction the alkyl groups are pointing) of 1.20 to 1.31 nm [42] versus that varying from about 1.55 to 1.73 nm in crystals of form I [48, 50]; the tighter inter-digitation seems to better stabilize the intrachain ordering in crystals of form II.

The above discussion concerning different crystalline forms of P3HT is important for understanding of structural transformation of P3HT crystals formed in PMMA matrix at different weight ratios of P3HT to PMMA. First, it has been found that the maximum position related to the (0-0) band in spin-coated P3HT-PMMA films experiences slight red shift at small P3HT to PMMA ratio, i.e., from 602 to 608 nm (Fig. 1a). Second, microscopy studies allowed us to distinguish two types of crystals in the blend samples, which have the short interplanar distances in the stacking direction (along the *b* axis of P3HT crystal) to be 0.417 (that is characteristic of ball-like structures, see Fig. 5a and Fig. 7) and 0.445 nm (characteristic of lamella structure shown in Fig. 5b), respectively. The latter value agrees well with the crystalline form II as discussed above, whereas the former one better corresponds to an intermediate form I’ reported by Roehling et al. [44], which possesses the interplanar distance of 0.41–0.42 nm. They also showed that the form I’ demonstrates an increase in the coherent domain size in the π − π stacking direction by a factor of ~ 2 (from 6.3 to 12.4 nm), as compared to form I in samples prepared from p-xylene, which can be responsible for the enhancement of the (0-0) band relatively to the (0-1) band in P3HT samples [50].

**Fig. 10:**
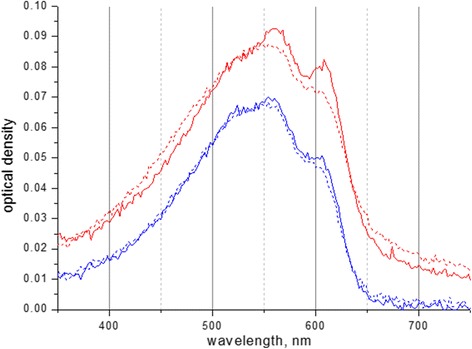
Electronic absorption spectra of as-prepared samples (*solid curves*) and the same samples 2 weeks later (*dotted curves*) of P3HT-PMMA (1:50 weight ratio, *red lines*) and P3HT-PMMA (1:4 weight ratio, *blue lines*)

Based on the above discussion, we can conclude that the composite P3HT-PMMA samples contain crystals of both forms of P3HT (I’ and II) because the interchain stacking distance varies from 0.42 to 0.44 nm for crystals of the different morphology. Thus, it can be suggested that the changing (0-0) to (0-1) ratio is related to the changing weight ratio of the different crystalline forms of P3HT, respectively, and the increasing (0-0) to (0-1) ratio most probably is due to increasing fraction of the form I’ in the blend, which promotes the increasing coherent domain size in the π − π stacking direction of P3HT domains. The reason of the above variations is tentatively assigned to hydrophobic forces acting on P3HT chains being in the polar environment, i.e., PMMA matrix, which forces P3HT aggregates to conform a specific arrangement inside the matrix. Such a process is most effective for smaller P3HT particles since the most influence is rendered onto the molecules being at the interface of P3HT-PMMA. Additional evidence that supports the above suggestion is the fact that the ratio of the first absorption maximum in respect to the sidebands decreases with time, which implies that the equilibrium between the different crystalline forms of P3HT in PMMA matrix evolves, namely, the form I’ gradually converts to the more thermodynamically stable form II (Fig. 10). Such a result reflects slow relaxation processes in PMMA matrix itself that acts on P3HT domains and thus renders a delayed effect which is more pronounced in samples with smaller P3HT particles.

## Conclusions

An increasing QY of PL which has been found in P3HT particles embedded into PMMA matrix is an unusual phenomenon since it takes place when the polymer molecules are still aggregated and where a strong exciton quenching should be normally observed. The increasing QY is assigned due to the two factors. The minor factor is the changing dielectric constant which facilitates a modest increase in QY by about 14%. The major factor is due to rearrangement of the polymer chains themselves. Better chain ordering in P3HT domains embedded into the PMMA matrix has been unequivocally proved by spectroscopy methods and calculation of the exciton bandwidth as well. The reason of the structural changes is tentatively assigned to hydrophobic forces acting on P3HT chains being in polar environment, i.e., PMMA matrix, which forces P3HT aggregates to conform a specific arrangement inside the matrix. Such a process is most effective for smaller P3HT particles since the most influence is rendered onto the molecules being at the interface of P3HT-PMMA. Particularly, it can be concluded that the composite P3HT-PMMA samples contain P3HT crystals of two forms, i.e., I’ and II, in which the interchain stacking distance varies from 0.42 to 0.44 nm. In form I’, intramolecular torsional disorder is reduced and most probably it promotes the increasing coherent domain size in the π − π stacking direction of P3HT domains, respectively. This is accompanied by the increasing first absorption maximum in respect to sidebands in spectra of composite P3HT films and by narrowing free exciton bandwidth, respectively. It is interesting to note that the narrowing exciton bandwidth is a factor which is responsible for increasing QY of PL emission in semiconductor nanoparticles as compared to the bulk crystals possessing wide energetic bands [51]. Narrow bands reduce smearing effect upon electronic transitions, thus facilitating more electrons falling to the same energy level. Thus, the observed enhanced QY of emission of P3HT particles can be interpreted in terms of the changing intermolecular packing and reduced intramolecular torsional disorder along with narrowing exciton bandwidth.
